# Random Force in Molecular Dynamics with Electronic Friction

**DOI:** 10.1021/acs.jpcc.1c03436

**Published:** 2021-06-27

**Authors:** Nils Hertl, Raidel Martin-Barrios, Oihana Galparsoro, Pascal Larrégaray, Daniel J. Auerbach, Dirk Schwarzer, Alec M. Wodtke, Alexander Kandratsenka

**Affiliations:** † Max-Planck-Institut für Biophysikalische Chemie, Am Faßberg 11, 37077 Göttingen, Germany; ‡ Institut für Physikalische Chemie, Georg-August-Universität Göttingen, Tammanstraße 6, 37077 Göttingen, Germany; § Université de Bordeaux, 351 Cours de la Libération, 33405 Talence, France; ∥ CNRS, 351 Cours de la Libération, 33405 Talence, France; ⊥ Universidad de La Habana, San Lázaro y L, CP 10400 La Habana, Cuba

## Abstract

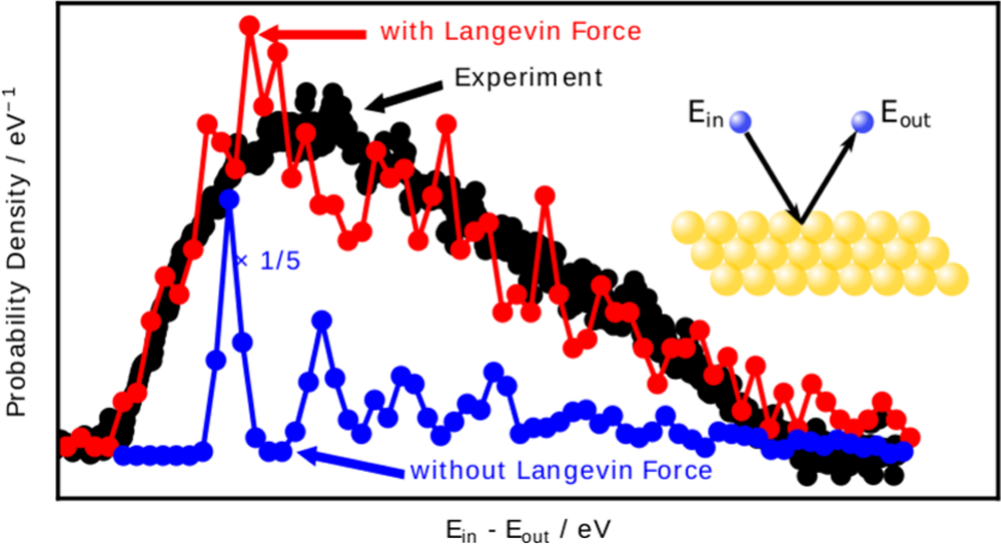

Originally conceived to describe thermal diffusion, the Langevin equation includes both a frictional drag and a random force, the latter representing thermal fluctuations first seen as Brownian motion. The random force is crucial for the diffusion problem as it explains why friction does not simply bring the system to a standstill. When using the Langevin equation to describe ballistic motion, the importance of the random force is less obvious and it is often omitted, for example, in theoretical treatments of hot ions and atoms interacting with metals. Here, friction results from electronic nonadiabaticity (electronic friction), and the random force arises from thermal electron–hole pairs. We show the consequences of omitting the random force in the dynamics of H-atom scattering from metals. We compare molecular dynamics simulations based on the Langevin equation to experimentally derived energy loss distributions. Despite the fact that the incidence energy is much larger than the thermal energy and the scattering time is only about 25 fs, the energy loss distribution fails to reproduce the experiment if the random force is neglected. Neglecting the random force is an even more severe approximation than freezing the positions of the metal atoms or modelling the lattice vibrations as a generalized Langevin oscillator. This behavior can be understood by considering analytic solutions to the Ornstein–Uhlenbeck process, where a ballistic particle experiencing friction decelerates under the influence of thermal fluctuations.

The Langevin equation originally served as an alternative to Einstein’s^1^ and Smoluchowski’s^2^ treatment of Brownian motion, the jittery back-and-forth hopping first seen under a microscope for pollen particles suspended in water that forms the physical basis for thermal diffusion. It explicitly describes time-dependent fluctuations seen in experiments with a random force derived using the fluctuation–dissipation theorem.^3^ The insights clarified by the random force helped to establish the molecular view of matter.^4^ Today, the Langevin equation is the most common theoretical ansatz used to model electronically nonadiabatic effects between atoms (or molecules) and solid metals.^5−9^ Here, nuclear motion takes the part of the Brownian pollen particle, and thermally fluctuating electron–hole pairs (ehp) of the metal play the role of the jiggling water molecules.

These frictional models of electronically nonadiabatic motion have broad applicability, for example, to describe the ion stopping power of metals,^10−14^ nonadiabatic dynamics^9,15−23^ like the thermalization of hot atoms,^20^ and even the mechanism of hydrogen atom adsorption to metal surfaces.^24,25^ Furthermore, a variety of approximations to compute the electronic friction tensor have been proposed.^8,14,17,26−36^ Experimental tests of these models are needed to determine which are most reliable.

Inelastic H-atom scattering from metal surfaces^24,25,37,38^ provides a direct probe of electronically nonadiabatic forces in a system that can be treated classically in full dimensions, including surface atom motion.^39,40^ Experimental and theoretical energy-loss distributions can be compared to test models of electronic friction. However, since the Langevin equation describes how a system evolves under the influence of a frictional drag and a random force, the experimental manifestations of a model of electronic friction cannot be realized without the influence of the random force. This poses the question how important is the influence of the random force?

When the Langevin equation is used to describe diffusion, the random force is essential, preventing motion from eventually coming to a standstill due to friction. However, to describe scattering and reactions of atoms and molecules at surfaces, its importance is not as obvious. In fact, the random force has often been ignored^9,15−23^ using as justification the fact that the projectile kinetic energy ϵ_0_ is much larger than thermal energy *k*_B_*T*. On the face of it, this assumption appears reasonable. For example, should we wish to describe ballistic motion of a H-atom in collisions with a metal, there is no danger of the system coming to a standstill and the timescale of a scattering collision can be very short, possibly rendering the ehp fluctuations unimportant.

In this article, we present molecular dynamics simulations using the Langevin equation to describe H-atom scattering from room-temperature metal surfaces, where the incidence energy is large and where interactions last only ≈25 fs. We compare these calculations to experimentally derived H-atom energy loss distributions.^24,25^ The trajectory simulations are generally in good agreement with the experiment provided the random force is included. However, neglecting it produces energy loss distributions that qualitatively fail to describe the experimental ones, even for ϵ_0_/*k*_B_*T* > 100. Only at surface temperatures below about 100 K does the influence of the random force diminish. This work shows that the physical mechanisms of nonadiabatic dissipation can easily be obscured by the random force.

To investigate the influence of the random force in the Langevin equation, we performed molecular dynamics (MD) simulations of H-atom scattering from two metals, Au and W. We compared outcomes employing two different approaches: model I,^39^ where the atom–surface interaction is described by a full-dimensional potential energy surface (PES) constructed by means of the Effective Medium Theory,^40−42^ and the surface is represented by a slab of metal atoms with periodic boundary conditions; and model II,^43,44^ where a three-dimensional (3D) PES produced by the Corrugation Reducing Procedure^45−47^ is used, and the surface is described by a generalized Langevin oscillator.^48−50^ In both models, the nonadiabatic coupling is described by the electronic friction coefficient depending on the background electron density (local density friction approximation).^14,17^ In model I, the background electron density appears self-consistently as it is necessary to calculate the energy; it depends on the positions of both projectile and surface atoms.^39^ In model II, one has to do additional *ab initio* calculations with the frozen surface to get the electron density as a function of a projectile position.^44^

The projectile is propagated by the Langevin equation of motion
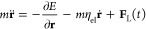
1where *E* is the potential energy of the system, *m* is the mass of the projectile, η_el_ is the electronic friction coefficient dependent on the system’s geometry, and **F**_L_(*t*) is the random force, which follows a Gaussian distribution with zero average and variance determined by the fluctuation–dissipation theorem^51^
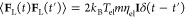
2where *T*_el_ is the temperature of the electron bath and **I** is the 3D unity matrix.

For both models, trajectories were run with an incidence energy of ϵ_0_ = 2.76 eV and an incidence angle of ϑ_i_ = 45° with respect to the surface normal. The azimuthal angles φ_i_ for the gold and tungsten calculations were defined with respect to the [101̅] direction and the [001] direction, respectively. Trajectories were initiated with the projectiles placed at random lateral positions 6 Å above the surface. The calculations were stopped after 1 ps or if the scattered projectile was found more than 6.05 Å above the surface.

Figure 1 shows the results using model I. The energy loss distribution constructed from the MD trajectories (•) that scatter into angles that match the angular acceptance of the experiment successfully reproduces an experimentally obtained energy loss distribution (◦). The scattered H-atoms exhibit a mean energy loss of approximately 1 eV and appear in a distribution with a remarkably broad width of 2.5 eV due to energy exchange with ehp and phonons. When *T*_el_ is set to 0 K, the MD simulations (■) fail spectacularly. Note that setting *T*_el_ = 0 K is equivalent to neglecting the random force.

**Figure 1:**
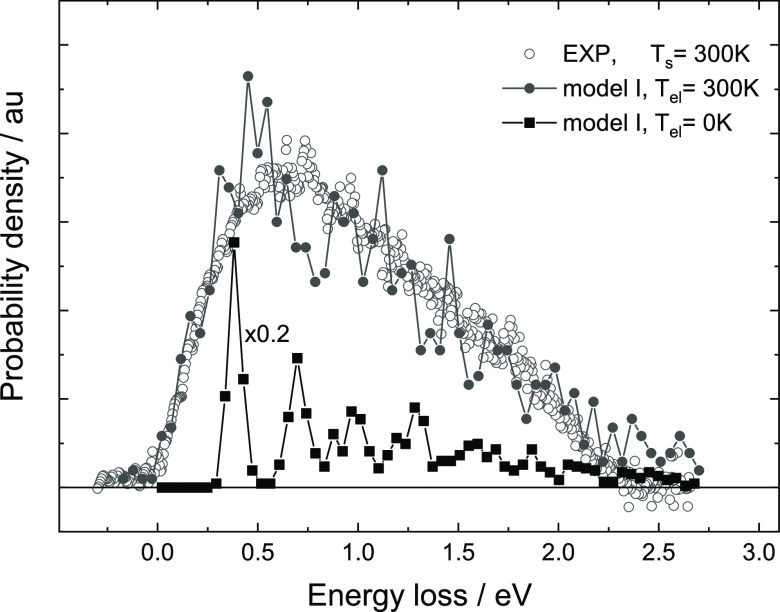
H-atom inelastic scattering from Au(111): comparing theory and the experiment. Using model I with *T*_el_ = 300 K (•), good agreement with the experiment (◦) is found. By setting *T*_el_ = 0 K, the random force is deactivated and theory (■) deviates from the experiment. For all three curves, ϵ_0_ = 2.76 eV, the phonon temperature is 300 K, ϑ_i_ = 45°, and ϑ_s_ = 45° with respect to the surface normal, while φ_i_ = 0° with respect to the [101̅] direction. Experimental results are taken from ref (24).

We show the influence of the random force on the energy loss distribution in Figure 2. Here, MD trajectories are generated as in Figure 1 using a PES with moving Au atoms (•), but *T*_el_ is varied between 300 and 0 K. As *T*_el_ decreases, peaks appear in the energy loss distribution. Analysis of trajectories reveals that these peaks correspond to “bounces”, that is, to interactions involving different numbers of collisions between H and Au atoms. The energy loss increases approximately linearly with each additional collision, reflecting the increased interaction time. Also shown in Figure 2 are two MD calculations employing a frozen surface (◦) with *T*_el_ = 0 and 300 K. For *T*_el_ = 0 K, peaks are even sharper than for the moving surface MD simulations at *T*_el_ = 0 K, the difference reflecting kinetic energy exchange between H and Au atoms. In contrast, at *T*_el_ = 300 K, it is hard to distinguish the energy loss distribution obtained when using a static surface approximation from that obtained when Au atoms are allowed to move.

**Figure 2:**
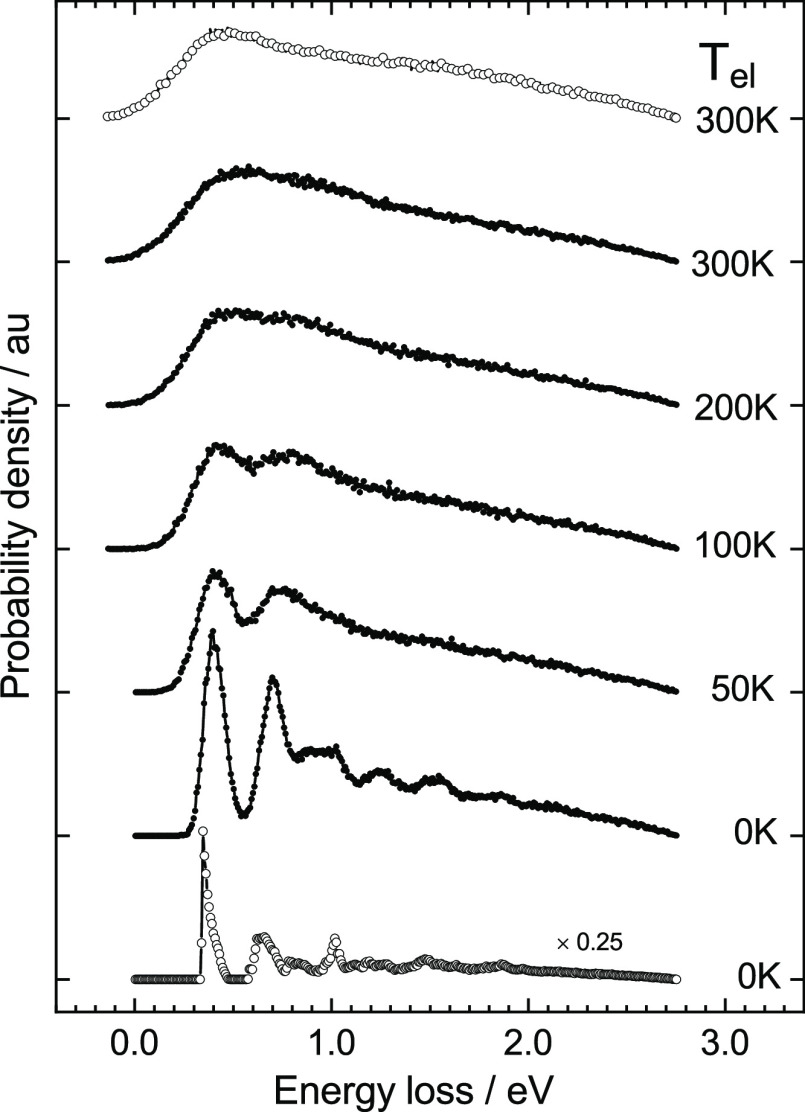
Electronic temperature determines the shape of the energy loss distribution. Energy loss distributions are shown for scattered H-atoms from a moving Au(111) surface with a phonon temperature of 300 K (•) and with a static lattice approximation (◦) at various electronic temperatures *T*_el_. Incidence conditions are the same as those in Figure 1; however, here, trajectories at all scattering angles are used.

Figure 2 also shows that the mean energy loss does not depend on the electronic temperature and is equal to 1.1 eV. The random force does not affect the sticking probability as well.

We next investigate the sensitivity of the energy loss distribution to the choice of the dynamical model. Figure 3a,b shows the results of MD simulations for H scattering from W(110) computed with models I and II. In Figure 3a, where *T*_el_ = 0 K, the two energy loss distributions are clearly distinguishable from one another. This is, however, not the case when *T*_el_ = 300 K (Figure 3b). Despite the moderate temperature and high H-atom incidence energy, it is clear that the broadening effects of the random force on the energy loss distribution smear out the differences in the scattering dynamics resulting from the two models.

**Figure 3:**
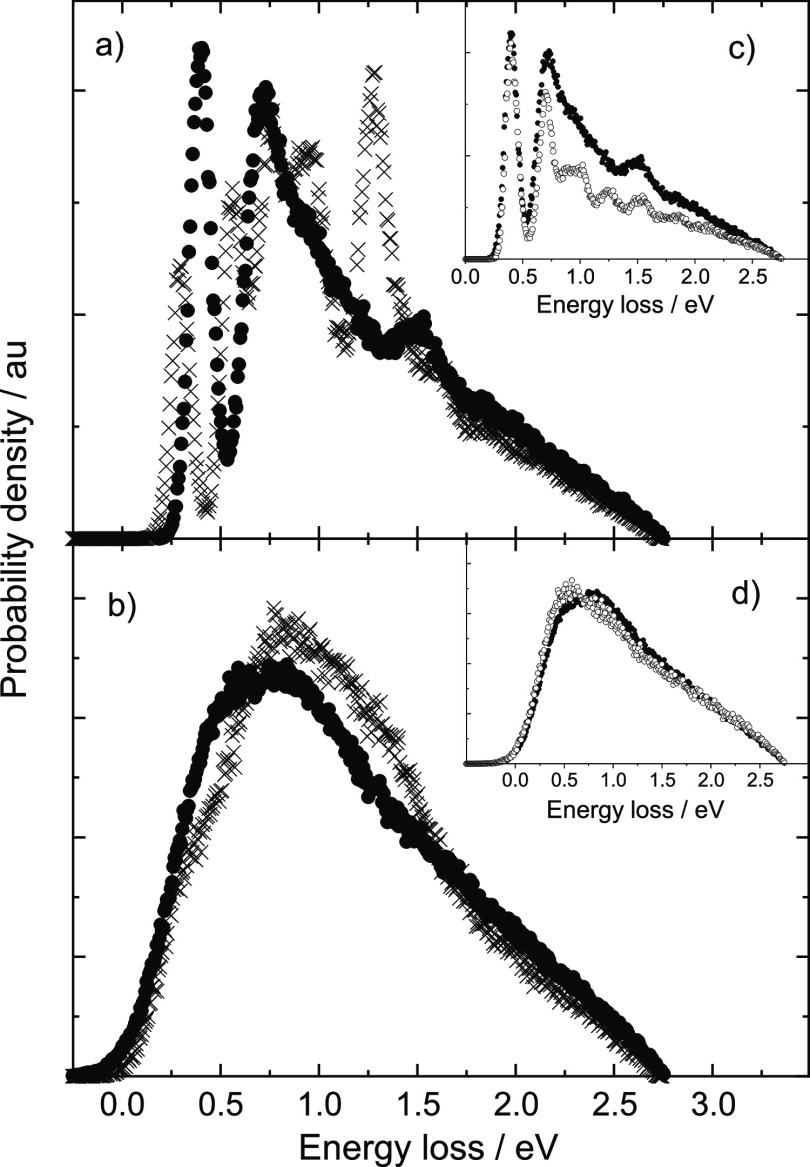
Obscuring influence of the random force at modest temperature: angle-integrated energy loss distributions for scattered H-atoms from W(110) using models I (•) and II (×) at (a) *T*_el_ = 0 K and (b) *T*_el_ = 300 K; in the insets, the energy loss spectra for H scattering from W(110) (•) and Au(111) (◦) are compared at (c) *T*_el_ = 0 K and (d) *T*_el_ = 300 K using model I. The phonon temperature in all cases is 300 K.

It is noteworthy that similar effects were observed in studying adiabatic dynamics of Ar and Xe at metal surfaces,^49^ where friction and fluctuating (random) force were due to the thermal bath of phonons. Here, the calculated properties (sticking coefficients, etc.) were not sensitive to the details of atom–surface interactions or changes in the phonon spectral density.

The sensitivity of the energy loss distribution to the identity of the metal is also interesting. To study this, we compared MD scattering results from two metals. Figure 3c,d shows comparisons of H scattering from fcc Au(111) (◦) and bcc W(110) (•), with both using model I. Remarkably, the energy distributions associated with these two metals can only be distinguished at low electronic temperatures.

To better understand the surprisingly strong influence of the random force on the width of energy loss distributions, consider a closely related problem that has an analytical solution: the one-dimensional motion of an ensemble of particles of mass *m* with incidence energy ϵ_0_ subjected to friction with characteristic deceleration time τ experiencing a random force at temperature *T*. This motion is described by the one-dimensional version of eq 1, where the conservative force (the first term in the right hand side) is omitted. The random force distribution is Gaussian with the second moment defined by eq 2, and the friction coefficient η_el_ = τ^–1^ is constant. This is known as the Ornstein–Uhlenbeck (OU) process,^52^ and we can use it to describe a scattering trajectory that has not reached equilibrium.

The ensemble’s initial velocity distribution is δ(*v* – *v*_0_); thereafter, it is normal, with the time-dependent expectation *v̅*(*t*) and standard deviation σ_*v*_(*t*) given by^53,54^
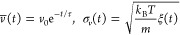
3where *v*_0_ = 2ϵ_0_/*m* is the initial speed of a particle and ξ(*t*) = 1 – *e*^–2*t*/τ^. Since the energy of the particle ϵ = *mv*^2^/2 is non-negative, its probability density function
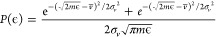
4has the form of a folded normal distribution^55^ with the mean energy
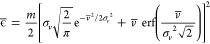
5and the energy standard deviation
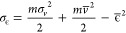
6

Substituting the mean velocity *v̅*(*t*) and the variance σ_*v*_^2^(*t*) for the OU process from eq 3 into eqs 4 and 6 using the definition of the cosine hyperbolic function, we derive the time-dependent energy distribution
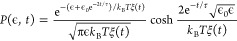
7and the corresponding standard deviation
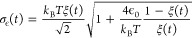
8

Figure 4a shows energy distributions from eq 7 for the one-dimensional OU process at ϵ_0_ = 2.76 eV and *T* = 300 K. Figure 4b shows the time-dependent width of the energy distribution eq 6 for four choices of ϵ_0_ and at *T* = 300 K. At *t* = 0, the energy distribution is a delta function. At an intermediate time, σ_ϵ_(*t*) overshoots *k*_B_*T*, reaching a maximum given by
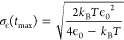
9where
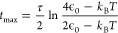
10before eventually falling back to the equilibrium value  in the limit of infinite time. Under the conditions of Figure 4a, *t*_max_ = 0.35τ, but σ_ϵ_(*t*) is much larger than *k*_B_*T* already at *t* = 0.1τ and remains quite broad until nearly completely decelerated.

**Figure 4:**
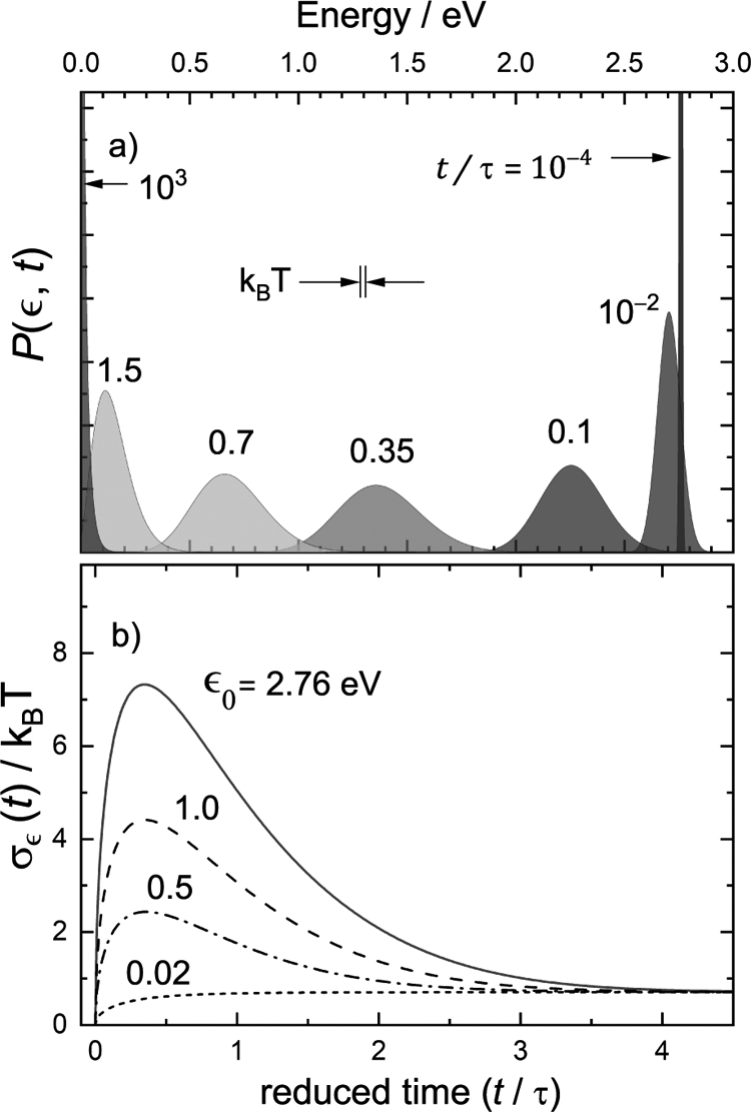
Time-dependent energy distribution of the Ornstein–Uhlenbeck process. (a) A particle with an incidence energy of ϵ_0_ = 2.76 eV decelerates under a frictional drag subject to thermal fluctuations at *T* = 300 K. Energy distributions are shown at various times, in units of τ, the characteristic time for deceleration. (b) The width of the distribution is shown for various choices of incidence energy ϵ_0_.

A naive view of eq 2 might suggest that because the distribution of random forces scales as , the width of the energy distribution scales similarly. However, when the random force introduces a thermally distributed change in velocity δ*v*, the resulting change in energy scales as (*v*_0_ + δ*v*)^2^ – *v*_0_^2^ = 2*v*_0_δ*v* + δ*v*^2^. The term 2*v*_0_δ*v* contributes to the energy distribution width in proportion to the hyperthermal velocity *v*_0_. Equation 9 shows this; σ_ϵ_(*t*_max_) scales as  for ϵ_0_ ≫ *k*_B_*T*. Furthermore, eq 10 shows that the thermal overshoot in the width of the energy distribution is absent only when ϵ_0_ < *k*_B_*T*/2 (see also Figure 4b). Clearly, for the OU process, one cannot justify ignoring the influence of the random force with an argument that ϵ_0_ is much larger than *k*_B_*T*. It is not then surprising that this argument is also incorrect when computing nonadiabatic MD trajectories in many dimensions.

The observations arising from our analysis of the H-atom energy loss distributions and of the OU process suggest that neglecting the random force for ballistic motion is generally unwise when considering the scattering properties more detailed than the mean energy loss or sticking probability. The results of this work also serve a warning. The generally good agreement seen between H-atom scattering experiments and MD simulations with electronic friction is due largely to broadening effects introduced by the random force. To experimentally distinguish different theories of nonadiabatic dynamics, experiments at low surface temperatures are needed. This could put new demands on theory as quantum dynamics may be important at low temperature.
